# Microbial Fuel Cell Biosensor with Capillary Carbon Source Delivery for Real-Time Toxicity Detection

**DOI:** 10.3390/s23167065

**Published:** 2023-08-10

**Authors:** Ademola Adekunle, Stefano Bambace, Fabrice Tanguay-Rioux, Boris Tartakovsky

**Affiliations:** 1 National Research Council of Canada, 6100 Royalmount Ave, Montreal, QC H4P 2R2, Canada; 2 Faculty of Engineering, McGill University, 3480 Rue University #350, Montreal, QC H3A 0E9, Canada

**Keywords:** biosensor, MFC, capillary action, potable water, toxicity, public safety

## Abstract

A microbial fuel cell (MFC) biosensor with an anode as a sensing element is often unreliable at low or significantly fluctuating organic matter concentrations. To remove this limitation, this work demonstrates capillary action-aided carbon source delivery to an anode-sensing MFC biosensor for use in carbon-depleted environments, e.g., potable water. First, different carbon source delivery configurations using several thread types, silk, nylon, cotton, and polyester, are evaluated. Silk thread was determined to be the most suitable material for passive delivery of a 40 g L^−1^ acetate solution. This carbon source delivery system was then incorporated into the design of an MFC biosensor for real-time detection of toxicity spikes in tap water, providing an organic matter concentration of 56 ± 15 mg L^−1^. The biosensor was subsequently able to detect spikes of toxicants such as chlorine, formaldehyde, mercury, and cyanobacterial microcystins. The 16S sequencing results demonstrated the proliferation of *Desulfatirhabdium* (10.7% of the total population), *Pelobacter* (10.3%), and *Geobacter* (10.2%) genera. Overall, this work shows that the proposed approach can be used to achieve real-time toxicant detection by MFC biosensors in carbon-depleted environments.

## 1. Introduction

Microbial fuel cell (MFC)-based biosensors have increasingly been researched in recent years for real-time detection of toxic compounds in water [1,2,3,4,5,6,7]. In anode-based MFC biosensors, the electrical current produced is based on the oxidation of organic matter at the anode by electroactive bacteria, leading to the production of electrons [6,8,9]. Carbon source oxidation at the anode is coupled with the oxygen reduction reaction at the cathode [10,11]. MFCs can have a dual-chamber configuration, where the anode and the cathode electrodes are placed in two different compartments, often separated by a membrane, or have a single-chamber configuration, where an air-breathing cathode is used [12]. For both configurations, the current produced by the MFC varies according to the activity of the microorganisms [1,6,13], which is influenced by several factors such as temperature, pH, conductivity, dissolved organics concentration, and the presence of toxic compounds [6,14,15,16,17,18].

MFC-based biosensors have been successfully used to measure the concentration of organic compounds in water through the measurement of the chemical oxygen demand (COD) and the biological oxygen demand (BOD) or to detect the presence of toxic compounds such as heavy metals, PCBs, pesticides, formaldehyde, etc. in water [1,2,3,4,5,6,7].

During toxicity detection, an increase in the COD concentration can lead to false readings from the biosensor since a higher concentration of carbon matter could mask the presence of toxicants by increasing electricity production [1,5,7]. To prevent this, it has often been reported that the biosensor could be operated with an external source of carbon to achieve carbon source saturation (C-saturation) conditions. However, operating the biosensor under C-saturation conditions is not necessary as long as the organic matter concentration remains stable, which is often the case for different water sources with low levels of biodegradable carbon sources, including tap water, rivers, and lakes. Nevertheless, a minimal organic matter concentration is required to enable toxicity detection since the current generated by the MFC needs to be sufficiently high to detect changes. For example, previous studies showed that a BOD concentration higher than 4–5 mg L^−1^ was needed in order to measure the BOD with a MFC-based biosensor [18,19]. Moreover, a higher organic matter concentration of the anolyte leads to an increase in the electricity generated [15]. At the same time, extremely low carbon source concentrations result in high biosensor sensitivity to the presence of toxicants and may lead to voltage reversal [2,3]. Deplete carbon environments thus need to be enhanced to increase the performance of the biosensor for water quality analysis.

Increasing the carbon content of the MFC could be difficult to achieve in remote environments where carbon source delivery by pump necessitates an external energy source. Another strategy recently proposed to increase the C-source supply to an MFC anode is the use of solid anolytes [20,21,22]. However, the use of solid organic sources in MFCs has been little studied and still faces several challenges [22]. Also, since the release of carbon from solid anolytes is driven by hydrolysis, it does not provide a constant dissolved organic matter concentration over time [22]. As such, a solution to provide a constant supply of carbon sources to an MFC sensor operating in remote areas is desirable. 

Recently, a cathode-based MFC biosensor has been proposed to overcome organic matter limitation [7,23]. However, the detection range and sensitivity of such cathode-based biosensing to toxic compounds need further investigation. Furthermore, the design could be complicated to integrate into existing water distribution networks. On the other hand, single-chambered MFC biosensors have been characterized thoroughly in several studies [10,19,24,25] and have a simple and more compact design [26]. If the organic matter limitation can be overcome, it would still be a better and cheaper alternative than any other MFC-based biosensor. 

In this study, we attempt to resolve the limitations associated with anode-based MFC biosensing in low-carbon source environments through passive delivery of a concentrated carbon source. In particular, we investigate a capillary-supported passive delivery system due to its low production costs and ease of reproducibility. The materials and configuration of the carbon source delivery system were first optimized prior to integration with the anode-based MFC biosensor system. Its detection performance was evaluated by detecting toxicity spikes in municipal tap water. Several toxic shock tests are conducted in order to demonstrate and evaluate the biosensing performance of the improved configuration. Overall, the study was designed as a proof-of-concept demonstration of the capillary system for controlled carbon source delivery and the biosensor’s capability as a real-time early warning (alarm) system for the detection of multiple organic and inorganic contaminants in water containing low concentrations of easily biodegradable organics, such as river, lake, and drinking water.

## 2. Materials and Methods

### 2.1. Stock Solutions and Analytical Methods

During start-up, the biosensors were fed with moderately hard water (MHW) to simulate an aquatic environment. The following MHW composition was used (in mg L^−1^): CaSO_4_ (60), MgSO_4_ (122.86), NAHCO_3_ (96), and KCl (4). All stock solutions were prepared using tap water with a conductivity of approximately 0.8 mS cm^−1^ and chlorine content of 30 µg L^−1^. Anolyte flow rate determinations of the examined biosensor configurations were carried out with tap water, while carbon source delivery rate was measured with a 40 g L^−1^ (as C_2_H_4_O_2_) stock solution of sodium acetate. After start-up, the influent stream was switched to tap water. To validate the biosensor’s performance for toxicant detection, the tap water stream fed to the biosensor was spiked with stock solutions of chlorine, formaldehyde, mercury, and cyanobacterial microcystins. The following influent stream concentrations of these toxicants were used: 3 mg L^−1^ (free chlorine) of sodium hypochlorite; 10 and 50 mg L^−1^ (as Hg) of HgCl_2_; 10 and 100 mg L^−1^ formaldehyde; and 3 µg L^−1^ of cyanobacterial microcystins.

Total chemical oxygen demand (tCOD) was measured using a PeCOD^®^ analyzer (MANTECH Inc., Guelph, ON, Canada). Free chlorine concentration in the tap water was measured using the HACH^®^ DPD (HACH, London, ON, Canada) free chlorine test kit. The viscosity of tap water at 25 °C was considered to be 0.893 g m^−1^ s^−1,^ and that of the acetate solution at 25 °C was considered to be 1.05 g m^−1^ s^−1^ [27]. Based on 5 measurements, standard deviation of these analytical methods was estimated to be less than 5% of the mean values.

### 2.2. Capillary Carbon Source Delivery Systems

Four different thread types (polyester, cotton, silk, and nylon) were tested to determine the best material for passive delivery of a carbon source. Two configurations based on the relative position of the carbon source reservoir (vertical or horizontal) were evaluated as described below.

**Figure 1:**
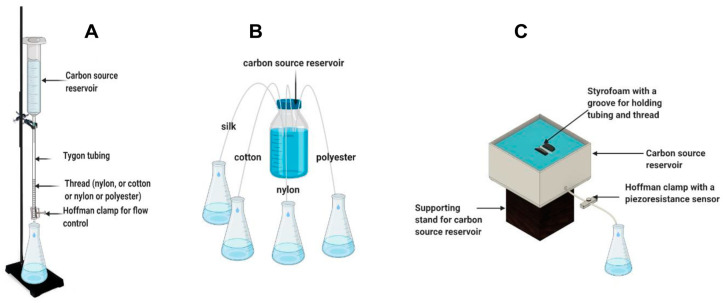
Illustration of thread-based capillary pump configurations with respect to the connection with a carbon source reservoir. Vertical connection with a carbon source reservoir (**A**), lateral connection with a carbon source reservoir (**B**), and modified lateral connection and flow control using a piezoresistance sensor–Hoffman clamp (**C**).

In the vertical reservoir–thread configuration, polyester, cotton, silk, or nylon threads were placed vertically and connected to a carbon source reservoir. Ten threads measuring 75 mm of the same type (five in the case of polyester due to its larger diameter) were encased in a Tygon tube (ID:1.6 mm). A 16 gauge by ½ inch needle attached to a 60 mL syringe was then inserted into the encasing tube of each thread type and tie-wrapped around the needle in order to maintain a seal. Figure 1A illustrates this thread-based delivery system and carbon source reservoir configuration. Two mm of each thread was allowed to hang out of the bottom of the tubing to relieve surface tension and prevent adhesion to the rim of the tubing. The thread–tubing combination was then passed through a small opening in a Parafilm-covered Erlenmeyer flask for liquid collection. A fifth Tygon tubing with no threads was used as a control. In the first part of the test, control of the flow rate of the capillary systems was achieved using a Hoffman clamp, where change in the flow rate was achieved by adjusting the clamp pressure using half-turns. Flow rate of each thread type was determined by measuring the weight change in the Erlenmeyer flasks collecting water. Overall, the vertical setup consisted of a total of 5 water reservoirs (vertically placed syringes without plungers) filled with the same volume of tap water (approximately 60 mL).

In the lateral reservoir–thread configuration, the four threads were immersed in a shared reservoir and passed through a Tygon tubing with no flow control (clamp), as shown in Figure 1B. In the first test, the reservoir was not refilled in order to evaluate the effect of changes in the head height on the flow rate. Furthermore, the flow rate was compensated for evaporation using a duplicate reservoir placed on a balance for constant measurement. After an initial evaluation, the lateral reservoir–thread configuration was slightly modified to increase flow rate. The Tygon tubing encasing the thread was slightly dipped into the carbon source, creating a siphon that increased the flow rate. Furthermore, in order to allow for a constant submersion of the tube and thread irrespective of the water reservoir level, the Tygon tube was attached to a groove on a 5 mm × 5 mm floating raft made of Styrofoam. A method for flow rate control was later introduced using a Hoffman clamp as in the vertical carbon source reservoir configuration. In addition, a piezoresistance-based pressure sensor was added to the clamp for more precise tuning of the flow rate. Figure 1C shows the final configuration of the lateral reservoir–thread carbon source delivery system.

### 2.3. Biosensor Design and Operation

**Figure 2:**
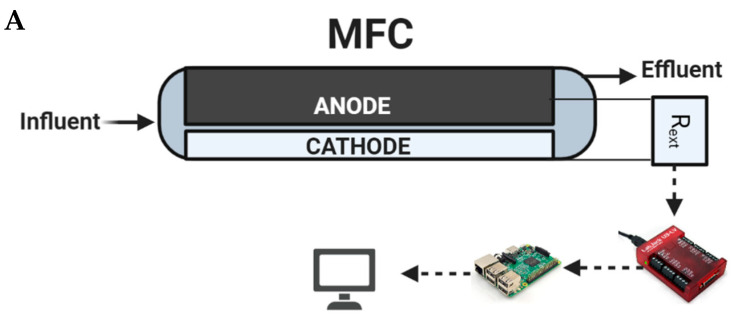

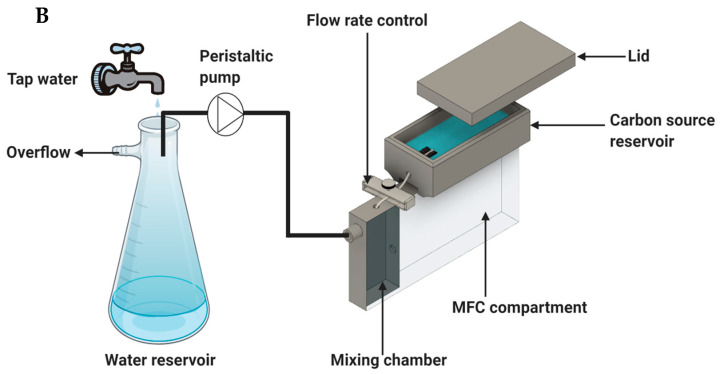
Schematic of the MFC compartment and measurement system (**A**) and tap water supply system, capillary pump, and MFC integration (**B**).

Two anode-based microbial fuel cell biosensors (denoted as S1 and S2), each connected to a passive carbon source delivery system, were simultaneously operated and used for tap water quality monitoring and toxicity spike detection. The third biosensor (S3) had an identical design and was continuously operated on moderately hard water and used for microbial population analysis. All biosensors were constructed according to a previously described design of a membraneless air–cathode flow-through MFCs [28], but the frames were 3D-printed acrylonitrile butadiene styrene (ABS) instead of nylon plates [29]. The anodes were made of double-layered carbon felt, each measuring 10 × 5 cm and 0.5 cm thick (SGL Group, Charlotte, NC, USA). The cathodes were made of 10 × 4 cm E4 electrodes containing manganese oxide catalyst (Electric Fuel Ltd., Bet Shemesh, Israel). Each MFC had a compartment volume of 100 mL. The biosensors were operated by a periodic connection to a 0.96 kOhm external resistor (R_ext_) in order to determine open-circuit voltage (*V_max_*) and closed-circuit voltage (*V_min_*) of the MFC. The data collection system and data collection algorithm used were described previously as “Algorithm-1” in [30]. *V_max_* (mV), *V_min_* (mV), and temperature (°C) values were measured at an interval of 15 min. To improve measurement accuracy, 5 consecutive voltage measurements obtained during each data acquisition cycle were averaged, resulting in less than a 10% standard deviation. A schematic diagram of the MFC, as well as the data acquisition and Rext control system consisting of Raspberry Pi single board computer and Labjack U3 DAQ device (Labjack Corp, Lakewood, CO, USA), is shown in Figure 2A.

After a determination of the most suitable carbon source delivery system (mostly based on such parameters as flow rate controllability and stability), it was integrated with the biosensor. The carbon source was delivered into a mixing chamber of the biosensor, where it was mixed with the influent water before reaching the anode compartment. To ensure a constant supply of fresh tap water, peristaltic pumps were used to deliver tap water to the biosensors from the top of an overflow reservoir with running tap water. An illustration of the integrated passive carbon source delivery system and biosensor is shown in Figure 2B. During toxic shock tests, the tap water reservoir was replaced with a bottle containing a prepared tap water solution spiked with a toxicant.

### 2.4. Microbial Community Analysis

The microbial community structure of the biosensor was examined by analyzing the electroactive biofilm developed on the carbon felt anode. DNA was extracted from the microbial inoculum (anaerobic sludge) and the carbon felt anodes (250 mg samples) with a Powersoil^®^ kit (MO BIO Laboratories Inc., Carlsbad, CA, USA) as described in an earlier study [30]. Quantification and purity assessment of the genomic DNA was carried out using a NanoDrop™ 1000 Spectrophotometer (Thermofisher Scientific, Waltham, MA, USA). Amplification was then performed with a HotStar polymerase master mix (Qiagen Inc, Toronto, ON, Canada). After the verification of the amplified DNA, it was purified and quantified with an Invitrogen kit (Thermofisher Scientific, Waltham, MA, USA). The fluorescence was then measured with a Tecan Safire microplate reader (Thermofisher Scientific, Waltham, MA, USA). Archaeal and bacterial 16S rRNA genes were sequenced using an Ion Torrent sequencing platform [31]. Taxonomic analyses (sequences treatments and analyses, OTUs generation, and taxonomic assignments) were carried out with an in-house developed 16S/ITS pipeline and were visualized in Microsoft Excel 2017 (Microsoft Corporation, Redmond, WA, USA).

## 3. Results and Discussion

### 3.1. Evaluation of Water and Carbon Source Flow Rates in the Passive Delivery System

Initial experiments were aimed at comparing different thread types for passive carbon source delivery. Accordingly, these tests were performed without connecting the passive delivery system to biosensors. Four different thread types (polyester, cotton, silk, and nylon) were evaluated to determine the material providing the most stable and controllable carbon source (acetate) flow rate. Initial experiments measured water flow rates in the four threads mentioned above and then extended to measuring sodium acetate flow for each thread. Since the tests were focused on comparing the long-term stability of carbon source flow in each thread, measurements were performed over time with average flow rates calculated for the entire time of each experiment.

**Figure 3:**
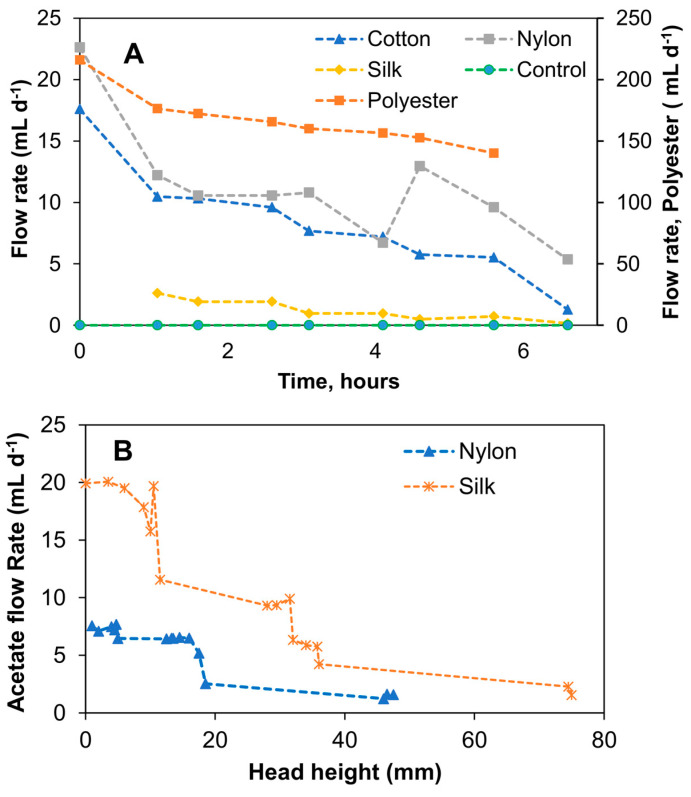
Capillary action aided flow rate test with different thread types connected to a vertical reservoir. Water flow rates using four different thread types and a no-thread control (**A**) and relationship between 40 g L^−1^ acetate flow rate and head height in nylon and silk threads (**B**). Standard deviation did not exceed 5% of the mean values.

As can be seen from the results shown in Figure 3A, the water flow rates in the threads connected vertically to the water reservoir tended to increase over time, apparently due to decreasing water levels. Nevertheless, averaged (during the experimental period) water flow rates were calculated to compare the flow rates. The following values were obtained: 167.4 ± 4.2 mL d^−1^ for polyester, 11.3 ± 4.6 mL d^−1^ for nylon, 2.1 ± 2.5 mL d^−1^ for silk, and 8.4 ± 4.2 mL d^−1^ for cotton; i.e., nylon and cotton threads showed comparable flow rates, while polyester thread flow was an order of magnitude higher. Silk thread demonstrated the lowest flow rate. These flow rates were used as an indication of flow rates that can be obtained using each thread type. Also, these measurements enabled preliminary estimations of the carbon source replenishing frequency and biosensor maintenance requirements. For sensor installation at a remote site, infrequent replenishment of the carbon source reservoir is desirable; accordingly, threads providing a slow and stable flow rate are preferable for MFC operation. As seen in Figure 3A, there was no flow in the control test (no thread), while positive flow rates were measured for all tested threads. In the following test, which used a concentrated solution of sodium acetate, due to an increased solution viscosity, the Hoffman clamp had to be adjusted (a 180° anticlockwise turn) to reduce resistance to liquid flow in the silk and nylon capillaries. Furthermore, due to the high flow rate in the polyester thread and the noticeable degradation of the cotton thread, these thread types were excluded from further testing. In particular, it was observed that the flow rate was affected by the increased liquid viscosity and the level of sodium acetate solution in the reservoir (i.e., distance from the liquid meniscus to the top of the syringe). The variations of these two parameters made the flow rate unstable and hard to control. As shown in Figure 3B, the flow rate declined over time, i.e., there was a correlation between the level of acetate in the reservoir and its flow rate in the silk and nylon threads. In a trend that was inconsistent with the previous water flow rate test, the average flow rate of acetate in the silk thread (9.9 ± 6.8 mL d^−1^) was higher than the acetate flow rate in the nylon thread (5.5 ± 2.2 mL d^−1^). It can be suggested that when using a vertical reservoir–thread configuration, the increased solution density led to an increase in the effect of gravity and, subsequently, to changes in the flow rates over time due to changes in the acetate levels. Based on this observation, it can be concluded that a vertical reservoir–thread configuration was not reliable for the proposed passive carbon source delivery system, as it did not provide stable flow rates over time.

**Figure 4:**
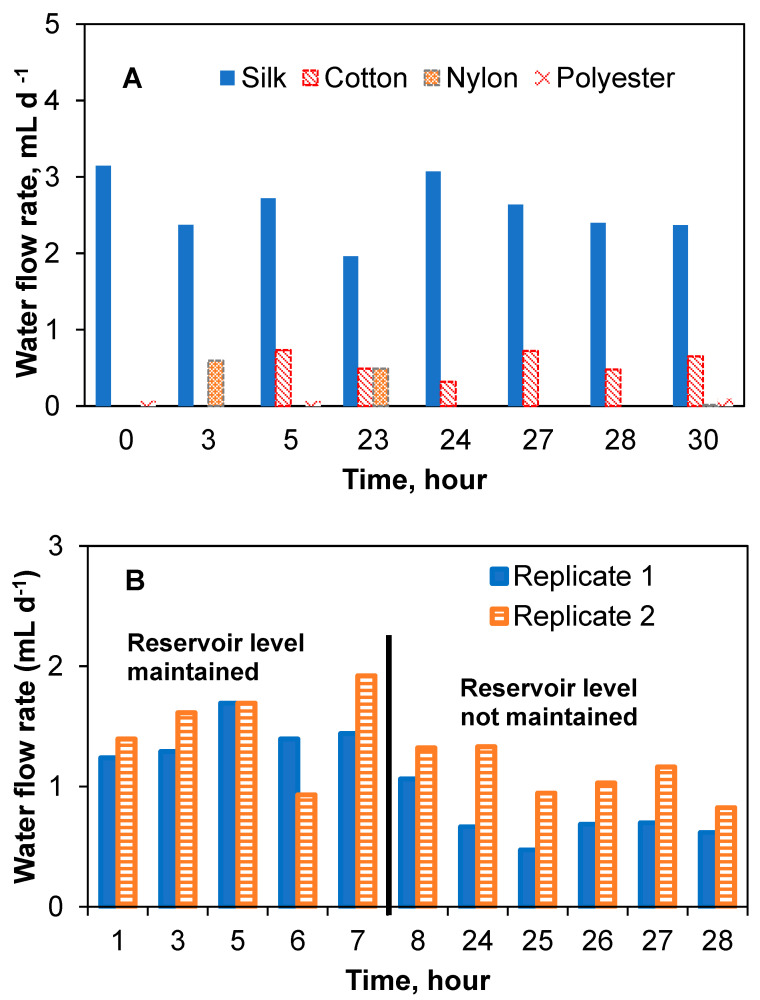
Capillary action aided flow rate test with different thread types connected to a lateral reservoir. Water flow rates for four different thread types (**A**) and replicate water flow rate tests with silk threads showing stability and possible effect of head height (**B**).

The flow rate determinations were repeated using the lateral reservoir design for the four thread types. Expectedly, due to the reduced effect of the gravitational force in the lateral reservoir–thread configuration, average flow rates were significantly lower than those achieved in the vertical reservoir–thread configuration, while the flow stability improved (Figure 4A). Average water flow rates (reservoir was refilled during this test) were 2.6 ± 0.4 mL d^−1^ (1.3 ± 0.5 mL d^−1^ in a duplicate test) for silk thread and 0.4 ± 0.3 mL d^−1^ for cotton thread, i.e., the lateral reservoir design resulted in significantly lower flow rates as compared to the vertical design. At the same time, more stable flow rates were achieved, as evidenced by reduced standard deviations. Nylon and polyester threads had negligible and inconsistent flow rates. Notably, polyester threads showed the highest flow rate in the previous experiment (vertical reservoir design). It can be suggested from the results that the wicking properties of both nylon and polyester threads were strongly dependent on the liquid level in the reservoir. Figure 4 shows the water flow rate of the threads in the lateral reservoir configuration. As can be seen in Figure 4B, the water flow rate in the two-silk thread setups in this configuration was fairly consistent over time (1.3 ± 0.5 mL d^−1^). The slightly lower flow rate shown in the later part of the replicate silk thread test was due to changes in the liquid level of the reservoir. A test with concentrated acetate solution showed that although the flow rate slightly decreased to 0.7 ± 0.4 mL d^−1^ and 0.5 ± 0.1 mL d^−1^ for the two silk thread replicates, respectively, as compared to tests with water, the flow rates were not statistically different. A summary of flow rates obtained for all tested thread materials and reservoir configuration is provided in Supplementary Materials.

Based on these observations, it was concluded that the lateral reservoir–thread configuration provides sufficient stability for sodium acetate delivery. In addition, it can be concluded that the silk thread is the best material among the materials evaluated, as it provided a stable and controllable flow of the acetate solution. Hence, a lateral carbon source reservoir–silk thread configuration was adopted for the following passive carbon source delivery experiments.

### 3.2. Passive Carbon Source Delivery to Biosensors

This section describes the results of capillary-based acetate delivery to biosensors. The silk thread-based passive carbon source delivery system was added to the MFC biosensors, as shown in Figure 2B. In this configuration, the biosensor influent stream (tap water) was amended with an acetate solution by the passive delivery system in the mixing chamber of the biosensor before reaching the anode. The COD concentration in this mixing chamber was periodically measured to confirm adequate carbon source delivery.

**Figure 5:**
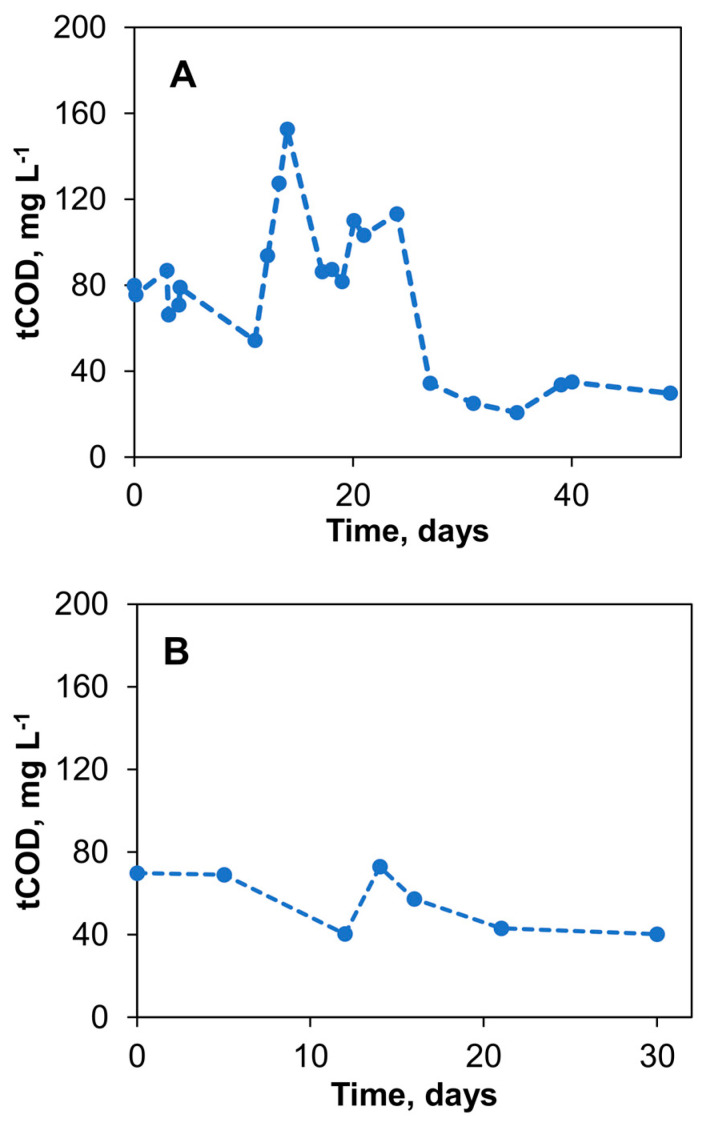
Passive amendment of organic matter concentration in tap water using a vertical reservoir– silk thread delivery system. Total chemical oxygen demand (tCOD) measured (**A**) and stabilization of flow rate through siphoning and elimination of head height effects (**B**). Standard deviation did not exceed 10% of the mean values.

Over 49 days of the initial biosensor experiments, the average flow rate of the 40 g L^−1^ acetate solution was 0.8 ± 0.4 mL d^−1^, leading to an average COD concentration of the tap water of 74.8 ± 34.5 mg L^−1^. Figure 5A shows the measured COD concentrations in the tap water stream after the delivery of acetate into the mixing chamber of the biosensor. The significant drop in COD observed after day 24 is consistent with the drop in the flow rate as the level of acetate in the reservoir decreases. Therefore, to minimize the effect of the carbon source level and stabilize the acetate flow rate during long-term biosensor operation using the passive delivery system, the delivery system configuration was further improved by placing the silk thread on a piece of Styrofoam, as described in Materials and Methods and shown in Figure 1C. The addition of a Styrofoam float ensures stable flow irrespective of changes in the level of the carbon source in the reservoir. Acetate flow control was achieved using a Hoffman clamp–piezoresistance sensor combination. Overall, through these adjustments, a more consistent COD concentration of 56.1 ± 14.8 mg L^−1^ was achieved in the delivery system over another 30 days of testing, as shown in Figure 5B. These changes decreased the coefficient of variation of the COD concentration measurements from 46.1% to 26.4%, highlighting the improved stability of the delivery method. As a result of stable acetate supply, the output voltage of S1 and S2 biosensors stabilized within two weeks from the start-up at 250–300 mV and 500–550 mV for *V_min_* and *V_max_* values, respectively.

### 3.3. Detection of Toxicity Spikes in Tap Water

Two biosensors (S1 and S2) with the integrated carbon source delivery system (Figure 2B) were used to observe changes in the sensor (MFC) voltage and current upon the appearance of toxicants in tap water. Spikes of chlorine, formaldehyde, Hg, and microcystins were introduced in tap water fed to these biosensors. These contaminants were chosen because they are used for water treatment (chlorine) or sometimes occur naturally (formaldehyde, Hg) [4,23,32,33,34,35]. Furthermore, the response to microcystins from *Microcystis aeruginosa* was also evaluated, as these compounds can be introduced into tap water distribution networks during cyanobacterial blooms [36]. Microcystins are very toxic at concentrations as low as 1 μg L^−1^ and can be difficult to detect [35]. During the shock tests, either one biosensor (S1 or S2) or both biosensors were used to evaluate a response to each toxicant, depending on the biosensor recovery from a previous toxic spike. The biosensors were only used if the measured open circuit (*V_max_*) voltage was deemed stable, showing less than 10% voltage variation during a 24 h period preceding the test. The open circuit voltage (*V_max_*) was observed to adequately reflect water toxicity, while the closed circuit voltage (*V_min_*) was shown to be linearly correlated with carbon source availability according to our previous results [3]. Each biosensor was operated for over 30 days prior to testing V_max_ response to the appearance of toxicants in the anode compartment liquid. Polarization tests (not shown) were carried out before the toxicants were introduced by changing R_ext_ in a range of 50 Ohm–10 kOhm, and showed a relatively high MFC internal resistance in a range of 1.6–2.0 kOhm. Such high internal resistance is typical for MFCs operated at low concentrations of biodegradable organics [2,3,16]. Furthermore, at least a 24 h recovery period was required after voltage measurements at low R_ext_ values (e.g., 50–500 Ohm). Accordingly, to avoid disruption of biosensor operation, the polarization tests were not carried out after each exposure to a toxicant.

**Figure 6:**
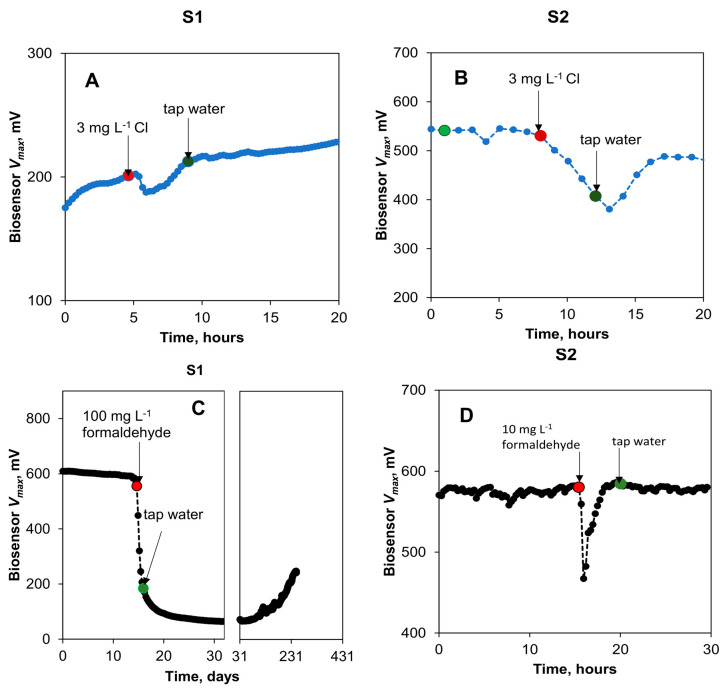
Biosensor (S1 and S2) response to toxic spikes in tap water with amended organic matter. Changes in open circuit voltage of the biosensors to spikes in chlorine (**A**,**B**) and formaldehyde (**C**,**D**) concentrations. Concentration changes are indicated by arrows and dots with red dot corresponding to formaldehyde introduction and green dot to formaldehyde removal from the influent stream. Time scale in panel (**C**) is given in days to demonstrate long-term biosensor recovery.

First, the biosensor response to a sudden increase in free chlorine was evaluated. In this test, during a period of 4 h, chlorine concentration in the influent stream of biosensors S1 and S2 was increased to 3 mg L^−1^, corresponding to two orders of magnitude of an increase over the background chlorine level found in tap water. Once the chlorine concentration increased, the *V_max_* of S1 and S2 biosensors almost immediately decreased, as shown in Figure 6A,B. During the test, the biosensor S1 inflow rate was approximately 30% lower as compared to S2 due to a technical problem. Accordingly, this biosensor received less water with high chlorine content, and a faster recovery of S1 was observed after the spike. Overall, the response of both biosensors to an increase in chlorine concentration showed that a spike can be successfully detected. It is important to emphasize that while several days were required for the signal stabilization after the increase in chlorine concentration, the biosensor reaction to the concentration spike was observed in less than 30 min after the spike. This delay can be attributed to both liquid transport to the anode chamber and toxicant diffusion through the carbon felt anode. It can be considerably reduced through optimization of the biosensor design (e.g., sensor miniaturization). It can be hypothesized that the addition of acetate to the tap water enabled the stable operation of the biosensors at low chlorine levels (30 µg L^−1^) typically present in tap water. Early detection of unintended Cl spikes in potable water is important in preventing known acute and chronic side effects of increased exposure to Cl at concentrations above 0.5 mg L^−1^, such as irritation in pulmonary, respiratory, and eye functions [37].

In the following test, the response of the biosensors to the presence of formaldehyde was tested by spiking the influent stream (Figure 6C,D). When exposed to 100 mg L^−1^ formaldehyde, the biosensor (S1) showed a rapid response (less than 30 min delay in detecting signal decrease) with an approximate 90% decrease in the estimated V_max_. This sharp response was observed during an exposure that lasted approximately 4 h. After reinstating the regular tap water flow, a slow recovery of the biosensor was observed (Figure 6C). This slow recovery can be attributed to the relatively slow re-growth of electroactive biofilm as well as to the slow removal of formaldehyde, which potentially accumulated in the biofilm, as demonstrated in previous MFC-based biosensor tests [23]. Another spike test with a lower concentration of formaldehyde (10 mg L^−1^) carried out with the second biosensor (S2) did not lead to a significant decrease in output voltages (Figure 6D). Previous studies have shown that the response of an anode-based MFC toxicity sensor is pronounced at concentrations greater than 50 mg L^−1^ [38,39], although in another study, a higher sensitivity limit of 0.1 mg L^−1^ was observed for an MFC operated without carbon source addition [40]. The initial drop in the voltage of S2 can be attributed to the introduction of air bubbles to the biosensor due to a technical error during the formaldehyde exposure test.

**Figure 7:**
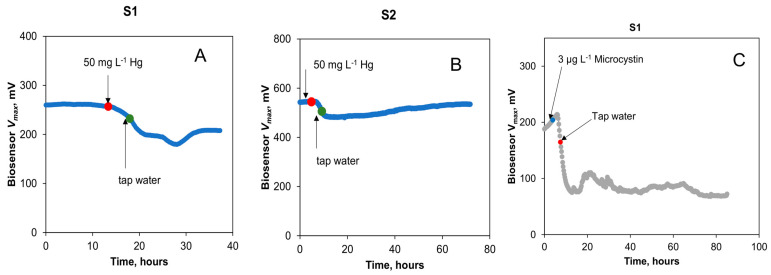
Biosensors S1 and S2’s response to toxic spikes in tap water with amended organic water. Changes in open circuit voltage of the biosensors to spikes in mercury (**A**,**B**) and cyanobacterial microcystins (**C**) concentrations. Concentration changes are indicated by arrows and dots. Standard deviation of analytical measurements did not exceed 5% of the mean values.

The impact of Hg on biosensor outputs was investigated in the next test. A spike in the concentrations of Hg to achieve 0.25, 1, and 10 mg L^−1^ in the influent stream led to no noticeable change in the output voltage of both biosensors. However, a brief exposure (3 h) to an increased Hg concentration of 50 mg L^−1^ led to a decrease in the V_max_ of both biosensors, as shown in Figure 7A,B. The decrease in the V_max_ values for S1 and S2 was 46% and 14%, respectively. The greater signal decrease for S1 can be attributed to the fact that it was not fully recovered from the earlier spike test with formaldehyde. Overall, the results of the Hg toxicity detection test are not unexpected, as previous studies have shown Hg to be either toxic to microorganisms or act as an electron acceptor in MFCs [25,41]. Importantly, this toxicity was detected in the tap water being monitored.

The final experiment was aimed at evaluating the detection of microcystins by the sensors. Microcystin presence in tap water was quickly detected by the biosensor (Figure 7C,D). The estimated *V_max_* of S1 decreased by 75% at a concentration of 3 μg L^−1^ of microcystins in the influent stream, demonstrating a high sensitivity of electroactive bacteria to microcystins. The microcystin concentration in this test was much lower than that of other toxicants tested in this study. Therefore, the MFC-based biosensor can be used for real-time detection of microcystins in drinking water. Notably, only the S1 biosensor was used in this test due to the drastic and prolonged toxicity of microcystins, while S2 was preserved for use in other tests. Nevertheless, S1 recovered 380 h after the exposure to microcystins. Importantly, a constant supply of acetate to the biosensor enabled toxicity measurements in tap water and biosensor recovery after electroactive bacteria exposure to a strong toxicant. Overall, it can be concluded that while the response of the two biosensors to an introduction of toxic compounds to the influent stream varied significantly, such variations are to be expected due to variations in microbial populations, biofilm density, and other factors affecting voltage produced by electroactive bacteria. Nevertheless, fast response (under 30 min) to toxicant appearance in the influent stream facilitates real-time qualitative detection of various toxicants.

### 3.4. The 16S Analysis of Microbial Community

Analysis of bacterial and archaeal 16S rRNA gene sequences was performed on the inoculum (anaerobic sludge) and on the anodic biofilm sample collected after 3 months of biosensor S3 operation. While S3 was also operated with periodic *R*_ext_ connection, toxicants were not added to the influent stream of this sensor, thus enabling direct assessment of microbial anodophilic populations developed during long-term operation on MHW. Since this biosensor was operated on MHW, the outputs were significantly lower (250–300 mV and 50–80 mV for *V_max_* and *V_min_* voltages, respectively) as compared with S1 and S2 sensors.

**Figure 8:**
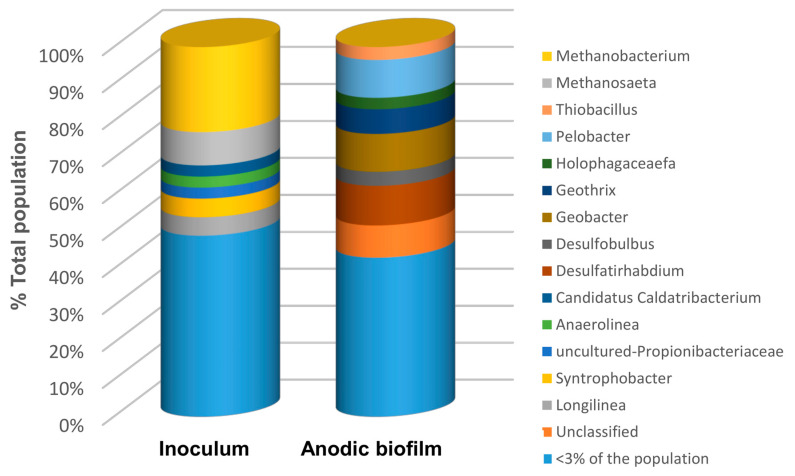
Bacterial and archaeal genera of microorganisms identified in anaerobic sludge (biosensor inoculum) and carbon felt anode of biosensor S3 after 3 months of operation.

Results of the 16S analysis shown in Figure 8 suggest that microbial populations of the inoculum were dominated mainly by *Archaea,* with *Methanobacterium* and *Methanoseta* spp making up 23% and 9%, respectively, of the population by relative abundance. *Methanobacterium* and *Methanoseta* are methane-producing organisms that are widespread in sewage and anaerobic sludge, which was used to inoculate the biosensors. Notably, *Methanoseta* utilizes acetate as its sole source of energy.

On the other hand, the microbial populations established on the anode of the biosensor S3 were distinctly different and dominated by the kingdom bacteria. In particular, the dominant genera (above a 5% threshold by relative abundance) included *Desulfatirhabdium* (10.7%), *Pelobacter* (10.3), *Geobacter* (10.2), and *Goethris* (6.9%). The presence of *Desulfatirhabdium*—an acetotrophic sulphate-reducing genera [42]—is explained by the presence of the sulphate salts in the MHW solution used for biosensor operation. *Pelobacter* are strictly anaerobic mixotrophic bacteria capable of autotrophy and heterotrophy, using either H_2_ and CO_2_ or saccharides to produce acetic acid. Furthermore, *Geobacter* spp. are known to proliferate in anaerobic electroactive biofilms [43]. The presence of the *Geobacter* genera can, therefore, be attributed to the availability of organic matter, albeit in low concentrations, from the possible by-products of the *Pelobacter* genera metabolism. In carbon limiting conditions achieved using the carbon source delivery system (biosensors S1 and S2), the prevalence of the *Geobacter* genera can also be expected. As would be expected, no acetoclastic archaeal populations can be found on the anode of the biosensor S3 due to very limited carbon source (acetate) availability. Overall, the results of 16S sequencing of biosensor S3 microbial populations show that an anodophilic biofilm with a significant presence of *Geobacter* can be developed even at an extremely low carbon source concentration. Although biosensor S3 was operated at extremely low carbon source concentration, the distribution of microbial populations at the anode is expected to be similar to S1 and S2 populations in the absence of toxicants. However, the introduction of toxicants to the influent stream is expected to significantly affect this distribution. Accordingly, a follow-up biomolecular study elucidating changes in anodophilic populations due to the presence of various toxicants might be required to develop procedures for rapid biosensor recovery after biofilm exposure to toxic compounds.

## 4. Conclusions

This study evaluated the performance of a passive capillary-based carbon source delivery system for anode-sensing MFC biosensors intended for real-time water quality monitoring under carbon-depleted conditions, e.g., as may be found in potable water. A lateral reservoir–silk thread setup was determined to be suitable for reliable passive delivery of sodium acetate to the biosensor. With the organic matter concentration in tap water maintained by this carbon source delivery system, an anode-sensing biosensor was successfully used to qualitatively detect the appearance of several contaminants, indicating the suitability of the proposed approach for potable water quality monitoring. Notably, the capillary system for carbon source delivery simplifies biosensor design and reduces energy consumption during biosensor deployment in remote locations. It can be concluded that the biosensor can be used as an alarm for the qualitative detection of acute, high-amplitude spikes in contaminants (a real-time early warning system). Furthermore, biosensor sensitivity to low concentrations of contaminants can be improved through the optimization of operating conditions. Overall, this study provides a proof-of-concept demonstration that anode-sensing MFC biosensors can be adapted for real-time detection of toxic spikes and long-term operation in carbon source-depleted conditions through organic matter supplementation.

## Data Availability

The data presented in this study are available on request from the corresponding author.

## Supplementary Materials

The following supporting information can be downloaded at https://www.mdpi.com/article/10.3390/s23167065/s1. Table S1: Summary of observed water flow rates (mL d^−1^) obtained with different thread materials and reservoir configurations.
