# Supplementary material for: Microbial Fuel Cell Biosensor with Capillary Carbon Source Delivery for Real-Time Toxicity Detection
Source: Sensors (Basel). 2023 Aug 10;23(16):7065. doi: 10.3390/s23167065 (PMC10458999; doi:10.3390/s23167065)
Supplement: Supplementary file 1 [file sensors-23-07065-s001.zip › sensors-2450083-supplementary.pdf]

Table S1. Summary of observed water flow rates (mL d^{-1}) obtained with different thread materials and reservoir configurations.

Thread material	Reservoir configuration	
	**Vertical**	**Lateral**
Polyester	167.4 ± 4.2	0.10 ± 0.07
Nylon	11.3 ± 4.6	0.12 ± 0.1
Silk	2.1 ± 2.5	2.6 ± 0.4
Cotton	8.4 ± 4.2	0.4 ± 0.3
Silk*	9.9 ± 6.8	n/a
Nylon*	5.5 ± 2.2	n/a

* Concentrated acetate solution used

n/a – not available
